# Predicting Anxiety and Depression Based on Video Game Addiction with the Mediating Role of Social Support

**DOI:** 10.62641/aep.v53i2.1745

**Published:** 2025-03-05

**Authors:** Zahra Jahanbakhshi, Nima Rezvani, Motahare Pourhasan, Shirin Ahmadi, Sayed Jafar Ahmadi

**Affiliations:** ^1^Faculty of Educational Sciences and Psychology, Shahid Beheshti University, 19839 69411 Tehran, Iran; ^2^Department of Psychology, School of Educational Sciences and Psychology, University of Mohaghegh Ardabili, 56199 11367 Ardabil, Iran; ^3^Psychology Faculty, Bard College, Annandale on Hudson, NY 12504, USA

**Keywords:** anxiety, depression, addiction to computer games, social support

## Abstract

**Background:**

Today, computer games have become one of the most popular forms of entertainment, especially among teenagers. While games may have various benefits, video games are shown to have different consequences for players, especially those who are younger, and can be highly addictive. The present research investigated the effect of computer game addiction on anxiety and depression in adolescents with the mediating role of social support.

**Methods:**

Overall, 304 adolescents aged 12–18 years old living in Tehran were included in the research through a convenient method. The required data were collected using the Trait-state Anxiety Questionnaire, Depression Scale, Social Support Questionnaire, and Computer Game Addiction Questionnaire and then analyzed using the structural equation model in AMOS software.

**Results:**

The results revealed that addiction to computer games had a significant effect on anxiety and depression. In addition, social support could act as a mediator in this relationship and reduce the harmful effects of computer game addiction on anxiety and depression.

**Discussion:**

According to the findings, to reduce anxiety and depression related to computer game addiction, it is necessary to pay attention to the improvement of social support for people through providing appropriate treatment plans, informing family and friends, and strengthening social connections and support networks.

**Conclusion:**

It is suggested that appropriate treatment programs be designed and implemented to reduce anxiety and depression in individuals with computer game addiction. These programs could include time management, behavior modification, enhancement of communication and social skills, and the provision of adequate social support through families, friends, and professional communities.

## Introduction

Nowadays, video games have become one of the most popular forms of recreational activity for all age groups, especially teenagers, worldwide [1]. The video game industry has extensively improved since the release of the first video game in 1972, which has led to the increasing engagement of different participation in this industry [2]. According to the available statistics, by the end of 2020, there were two billion and seven hundred million gamers worldwide [3]. While games may have a wide range of advantages, it has been shown that computer games can have different consequences for players, particularly younger ones such as teenagers [4, 5]. By changing their behavioral patterns, they weaken the individual’s will and authority to the point where they prefer the game over other activities and become addicted to playing computer games [6].

Addiction to these kinds of games can have various negative consequences for teenagers, including academic failure, reduced mobility [7], stress [8], aggression [9], and the emergence of suicidal thoughts [10]. Anxiety and depression are the other consequences of addiction to computer games. Currently, mental disorders affect 14% of the world’s population, with anxiety disorders and depression being the most common disorders, affecting 284 and 264 million people worldwide, respectively [11].

In the relationship between video games and anxiety and depression, it is important to mention that engaging in some other games can threaten various aspects of a person’s mental health [12]. Social support is one of the factors through which computer game addiction may affect people’s mental health and make them susceptible to anxiety and depression. Social attachments play an important role in maintaining mental health [13].

Research [14] has shown that people who become addicted to the Internet and its various dimensions, including games, may become more distant and isolated from the community. Social support acts like a shield, keeping a person safe from mental illness [15].

Weaknesses of the conducted research include the lack of an exact causal relationship between anxiety and depression and computer games. It is unclear whether computer games lead to anxiety and depression or if people with anxiety and depression are more susceptible to this addiction [16]. Another point is the contradiction between the results, as some studies have shown that gaming addiction leads to anxiety and depression, while others have not proven such a relationship [17, 18, 19]. The present study is not a longitudinal study but a cross-sectional study. Therefore, a definite relationship cannot be taken from it.

Addiction to these kinds of games can have various negative consequences for teenagers. In this regard, the current study aims to investigate the effect of computer game addiction on the formation of anxiety and depression with the mediating role of social support among Iranian teenagers.

## Materials and Methods

This research is practical in terms of purpose and descriptive in terms of method. The structural equation model analyzed the data in two steps. First, confirmatory factor analysis of the variables was checked, and after confirmation, the type of mediation model was determined by the Barron and Kenny method. The statistical population included all adolescents aged 12–18 years old in Tehran, and 329 adolescents were included in the study using the available convenient sampling method. The minimum sample size in structural equations is 200 people; therefore, more samples were included in the research for reliability and to ensure a lower error rate [20].

Three high-traffic game clubs were selected from three points in the north-south and the center of Tehran. After visiting the clubs, an informed consent form and a link to the questionnaire were sent to the parents, and they were requested to ask their children to complete the questionnaire if they were satisfied. The inclusion criteria included being a student and showing a willingness to participate in research. These questionnaires were designed on the Porsline website and made available to the subjects online by sharing their links in class groups and on social networking platforms such as Telegram channels and WhatsApp groups to facilitate easy access for students who wanted to participate in the research and answer the questions.

The ethical approval code for this study is IR. UMA.REC.1401.080, which the Ethics Committee in Research has approved at the University of Mohaghegh Ardabili.

### Measurements

Spielberger’s State-Trait Anxiety Questionnaire: This questionnaire was created in 1971 by Spielberger *et al*. [21]. It contains 40 questions that focus on state anxiety (20) and trait anxiety (20). Questions related to state anxiety are scored on a four-point Likert-type scale, implying not at all, sometimes, generally, and very much. The questions associated with trait anxiety are scored in the same way with 4 options (rarely, sometimes, most of the time, and almost always). Finally, two scores are obtained, indicating state anxiety and trait anxiety. Each person can gain a score between 20 and 80 in these two types of anxiety. Spielberger *et al*. [21] reported the Cronbach’s alpha coefficient of the state anxiety and trait anxiety scales as 0.92 and 0.90, respectively. He also reported that the retest coefficients of the state anxiety scale were 0.16 to 0.86 and that Cronbach’s alpha coefficient was 0.94 [21]. In the present study, reliability was obtained using the internal consistency method by calculating Cronbach’s alpha coefficient for the total depression score, which was 0.84.

Kutcher Depression Scale: It was created by LeBlanc *et al*. [22] to measure the severity of depression symptoms in adolescents. It is a self-report tool that has eleven items. Each statement requires the subject to answer the questions using four Likert-type scale options based on the mental states experienced in the last week. Scoring on this scale is direct; the subject’s score can vary between 0 and 33. This test also includes two subtests, namely, depression and suicidal thoughts. In the present study, reliability was obtained using the internal consistency method by calculating Cronbach’s alpha coefficient for the total depression score, which was 0.84.

Farhadi Computer Game Addiction Questionnaire: This questionnaire, which was compiled by Soltani and Farhadi (2017) [23], contains 13 items. All the items of this instrument are based on a 5-point Likert-type scale (4 = always, 3 = often, 2 = sometimes, 1 = rarely, 0 = never), and the range of scores in this questionnaire is between 0 and 52. A higher score indicates a greater addiction to computer games and vice versa. In the research by Soltani and Farhadi [23], to estimate the validity of the questionnaire, it was first implemented on 98 or more people, and the binomial method and Cronbach’s alpha were used. After halving the computer game addiction questionnaire questions and calculating the scores for each half, the correlation coefficient value between the scores obtained from halving was 0.77. Using the Spearman-Brown method, the reliability coefficient was 0.87. The reliability coefficient obtained with Cronbach’s alpha internal correlation method was also 0.90, indicating high reliability [23]. In the present study, reliability was obtained using the internal consistency method by calculating Cronbach’s alpha coefficient for the total score of computer game addiction, which was 0.90.

VAX Social Support Questionnaire: It was developed in 1960 based on Cobb’s definition of social support [24]. It is also known by other names, such as VAX Social Support and Phillips Social Support. The SS-A questionnaire has 23 questions, and the answer to each question follows a five-point Likert-type scale ranging from very little to very much. This questionnaire measures the level of social support individuals receive from their families, friends, and others. In the present study, reliability was obtained using the internal consistency method by calculating Cronbach’s alpha coefficient for the total social support score, which was 0.86.

### Data Analysis

After entering the data into SPSS 26.0 statistical software (Armonk, NY, USA), produced by IBM, and removing outlier data using Z standardization (where observations with an absolute value of Z greater than 3 were considered outliers), 304 questionnaires were entered into statistical analysis. The data collected in this research were analyzed using SPSS 26 and AMOS 24.0 statistical software (Armonk, NY, USA), produced by IBM, software, employing descriptive statistics (means and standard deviations) and structural equation modeling.

The obtained data were analyzed using structural equation modeling. First, three assumptions of data analysis—normality, outliers, and missing data—were checked. The Mahala Nobis index was used to detect outlier data, and the results indicated that there were no outliers or missing data.

The confirmatory factor analysis of research variables was examined in the first stage of data analysis. The hypothesized structural model included the predictor variable (addiction to computer games), the mediator variable (social support), and the criterion variables (anxiety and depression). The hypotheses were tested after confirming the final fit of the research model. Finally, the type of the mediation model was determined by the method described in previous research [25].

## Results

Overall, 304 people (M = 15.47, standard deviation (SD) = 1.6) participated in this research. The demographic characteristics of the participants are reported in Table 1.

**Table 1.** **Frequencies of demographic variables**.

Variable	Valid	Frequency	Percentage (%)
Gender	Men	256	84.2
	Women	48	15.8
Age (year)	12.00	15	4.9
	13.00	19	6.3
	14.00	42	13.8
	15.00	79	26.0
	16.00	71	23.4
	17.00	36	11.8
	18.00	42	13.8

In the analysis of the structural equation model, the condition of normal distribution of data is established when the numerical value of Skewness and Skewness is between ±2 and ±3, respectively [26]. Based on the results, the data had a normal distribution (Table 2).

**Table 2.** **Mean, SD, Skewness, Kurtosis, and correlations**.

Variable	Mean	SD	Sk	Ku	Correlations								
					1	2	3	4	5	6	7	8	9
Addiction to computer games	24.50	14.24	0.52	0.04	1								
Depression	7.78	5.94	0.72	0.01	0.34^**^	1							
State anxiety	42.07	10.98	0.46	0.11	0.27^**^	0.79^**^	1						
Trait anxiety	42.23	11.96	0.35	–0.13	0.29^**^	0.70^**^	0.88^**^	1					
Anxiety	84.30	22.26	0.43	–0.01	0.29^**^	0.74^**^	0.96^**^	0.97^**^	1				
Family support	25.23	4.02	–0.35	0.07	–0.28^**^	–0.23^**^	–0.33^**^	–0.29^**^	–0.32^**^	1			
Friends support	24.78	5.16	–0.35	0.04	–0.20^**^	–0.27^**^	–0.35^**^	–0.30^**^	–0.33^**^	0.43^**^	1		
Other support	21.43	3.40	0.30	0.25	–0.18^**^	–0.10	–0.15^**^	–0.11^*^	–0.13^*^	0.42^**^	0.59^**^	1	
Social support	71.45	10.26	0.02	0.22	–0.27^**^	–0.26^**^	–0.35^**^	–0.30^**^	–0.34^**^	0.75^**^	0.87^**^	0.79^**^	1

Note. SD, standard deviation; Sk, Skewness; Ku, Kurtosis. ^**^ The correlation is significant at the 0.01 level (2-tailed). ^*^ The correlation is significant at the 0.05 level (2-tailed).

The correlation between the research variables, such as the negative relationship between social support and depression (–0.26) and the positive relationship between computer game addiction and anxiety (0.29), was consistent with the background (Table 2). Discriminant validity examines the difference between one construct and another, implying that two constructs do not measure the same subject and are different. According to this criterion, the correlation between the two constructs should not be more than 0.9. According to the results (Table 2), all research variables had discriminant validity.

The first step is the confirmatory factor analysis for each variable, which evaluates the relationship between latent and observed variables. According to previous research [27], items with a loading factor less than 0.3 or negative are excluded from the analysis. The factor loadings of items 13 and 10 of the social support were less than 0.3 and were excluded from the analysis.

In the second stage, the structural model was evaluated to test the significance of the research hypotheses and estimate the coefficient of determination. The fit of the model is confirmed if at least three indicators meet the conditions [27]. The fit indices of the structural model presented in Table 3 (normalized chi-square (CMIN/DF) = 2.43, goodness of fit index (GFI) = 0.9, comparative fit index (CFI) = 0.91, and root mean squared error of approximation (RMSEA) = 0.07) demonstrate that the model has a good fit.

**Table 3.** **Fitness indicators**.

Index	CMIN/DF	CFI	GFI	RMSEA
Value	2.43	0.91	0.9	0.07
Acceptable	<5	>0.9	>0.9	>0.3, <0.9

Note. CMIN/DF, normalized chi-square; CFI, comparative fit index; GFI, goodness of fit index; RMSEA, root mean squared error of approximation.

### Testing Research Hypotheses

Based on the findings (Table 4), the relationships between addiction to computer games and social support (β = 0.35, *p* < 0.05), social support and anxiety (β = –0.50, *p* < 0.05), social support and depression (β = –0.38, *p* < 0.05), addiction to computer games and anxiety (β = 0.19, *p* < 0.05), and addiction to computer games and depression (β = 0.29, *p* < 0.05) became significant, and research hypotheses were confirmed.

**Table 4.** **Path coefficients of the proposed model**.

Paths	Β	Standard error	Sig.
Addiction to computer games → Anxiety	0.19	0.08	<0.05
Addiction to computer games → Depression	0.29	0.6	<0.05
Addiction to computer games → Social support	0.35	0.07	<0.05
Social support → Anxiety	–0.50	0.15	<0.05
Social support → Depression	–0.38	0.10	<0.05

Note. Sig., level of significance.

### Mediation Model Type

The mediation type of social support was determined according to the method presented in previous research [25]. In this method, the path coefficient between independent and dependent variables is compared with the presence of the mediator variable (the full mediation model) and without the presence of the mediator variable (the direct model).

**Fig. 1.**
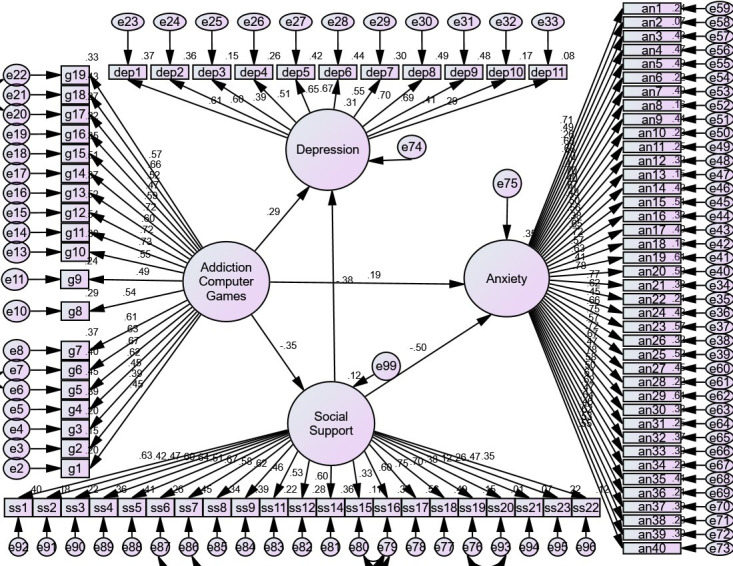
**Direct Model**. Note. The indirect paths between computer game addiction and anxiety and depression are considered to be zero, and the coefficient of direct paths between computer game addiction and anxiety (β = 0.19, *p* < 0.05) and depression (β = 0.29, *p* < 0.05) were obtained.

A path coefficient was obtained between computer game addiction and anxiety (β = 0.19, *p* < 0.05) and depression (β = 0.29, *p* < 0.05) in the direct model (Fig. 1).

**Fig. 2.**
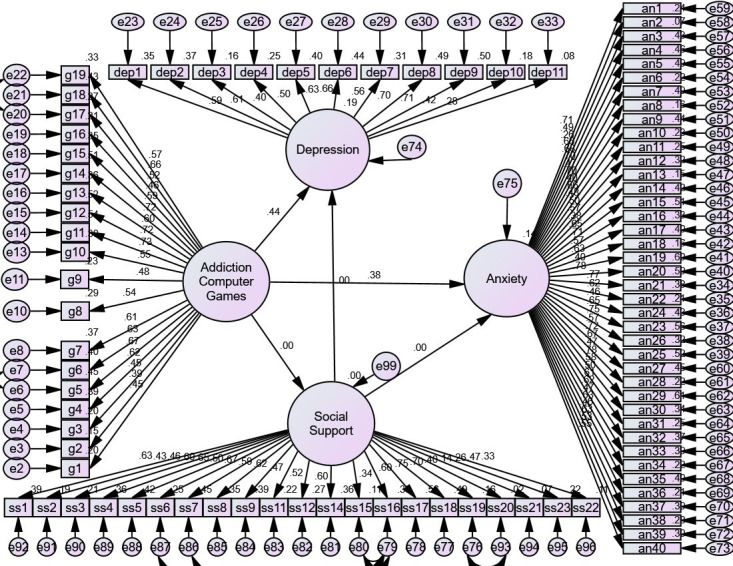
**Full Mediation Model**. Note. No path is considered zero, and path coefficients between computer game addiction and anxiety (β = 0.38, *p* < 0.05) and computer game addiction and depression (β = 0.44, *p* < 0.05) were obtained.

A path coefficient was obtained between addiction to a computer game and anxiety (β = 0.38, *p* < 0.05) and depression (β = 0.44, *p* < 0.05) in the full mediation model (Fig. 2).

In the comparison between the direct and full mediation models and the reduction of the path coefficient in the full mediation model, it was concluded that social support plays a partial mediation role.

## Discussion

The present study investigated the effect of computer game addiction on anxiety and depression, with a focus on the mediating role of social support. The results of statistical analysis indicated that there was a significant relationship between anxiety, depression, and addiction to computer games, with social support serving as a mediator. These findings are consistent with those of previous research [28, 29, 30, 31, 32], demonstrating that excessive engagement and addiction to computer games can be associated with various negative psychological consequences, including anxiety and depression.

In explaining this relationship, it can be concluded that addiction to computer games may lead to social isolation and a decrease in social support. The more an individual engages in computer games, the more they may become separated from the real world and their community, neglecting a person’s personal and daily life [13, 33, 34]. Social support has important functions, such as providing psychological stability and acting as a shield against stressful events. It also helps individuals feel loved, cared for, valued, and respected. With the reduction of social support, people’s resilience against psychological and environmental pressures decreases, leading to a decrease in life satisfaction and depression and anxiety [19, 35].

On the other hand, when a person has less social support, they become more vulnerable to various psychological damages, including anxiety and depression. The current research has some limitations. One of the main limitations was that the majority of our participants were boys. Therefore, investigating computer game addiction and its role in the formation of anxiety and depression with a larger number of female samples could be important. Additionally, the present study is cross-sectional, so it was impossible to determine the exact causal relationships between the variables. Future research should employ more detailed methods like longitudinal studies to determine causal relationships between these variables better. The greater the support individuals receive from families, friends, and others, the better they can cope with environmental pressures, reducing game addiction [19]. Therefore, it is suggested that future studies use social support as a moderator.

Another limitation of this study was the use of self-report measures without clinical interview conditions. Future studies could explore other factors that play a role in the relationship between game addiction and psychological problems, such as self-esteem, emotion regulation, and coping styles. Separating the genres of games in future research is also crucial, as it would allow for an examination of the specific harmful effects of different game genres.

## Conclusion

Based on the findings of this study, it is suggested that appropriate treatment programs be designed and implemented to reduce anxiety and depression in individuals with computer game addiction. These programs could include time management, behavior modification, enhancement of communication and social skills, and the provision of adequate social support through families, friends, and professional communities. Additionally, increasing public awareness about the psychological and societal impacts of computer game addiction is essential. Education and awareness for families, educators, and researchers on recognizing and managing computer game addiction can be effective in preventing and treating this issue.

Finally, creating healthy and supportive social environments is critical for improving the psychological well-being of individuals with computer game addiction. This includes eliminating isolation, forming support groups, providing social opportunities, and offering constructive ways to share diverse interests and activities.

## Availability of Data and Materials

Data are available on reasonable request from the corresponding author.
